# Absorbent Cotton

**Published:** 1881-04

**Authors:** Frank L. Slocum


### ABSORBENT COTTON.

Absorbent cotton has in the last few years taken such an important place among the necessities and conveniences of the pharmacist and physician, that it is very desirable for the pharmacist to be able to procure or himself manufacture it. The manner in which it is prepared is kept secret, and there is no literature on the subject up to the present time, it is believed.

At the request of Prof. Maisch some experiments have been made, the following process yielding the most satisfactory results.

Take of the best quality of carded cotton batting any desired quantity and boil it with a desired solution of caustic potassa or soda for one-half hour, or until the cotton is entirely saturated with the solution, and the alkali has saponified the oily matter. Then wash thoroughly, to remove all soap and nearly all alkali; press out the excess of water, and immerse in a 5 per cent. solution of chlorinated lime for 15 or 20 minutes; again wash, first with a little water, then dip in water acidulated with hydrochloric acid, and thoroughly wash with water, and again boil for 15 or 20 minutes in a 5 per cent. solution of caustic potassa or soda; now wash well, dipping in the acidulated water and washing thoroughly with pure water. Afterwards press out and dry quickly.

Boiling with caustic alkalies saponifies all oil and greasy matter, and the soap formed is removed by washing; if it were not washed out before bleaching, an insoluble lime soap would be formed and precipitated on the fibres. Dipping in very dilute dydrochloric acid after bleaching renders the removal of calcium compounds more easy. At this stage the cotton has all the appearance of absorbent cotton, but it absorbs water rather slowly; when again boiled with a caustic alkali, more organic matter of a non-greasy nature is removed, and the cotton is rendered perfectly absorbent. On dipping the cotton in dilute hydrochloric acid at the last washing a chloride is produced with the caustic base used, which is more readily washed out than the alkali itself.

The amount of loss by this process is practically 10 per cent.

A sample of 360 grains lost, on boiling with alkali and bleaching, 15 grs., or 4.17 per cent., and 270 grs. of this bleached sample lost, on again boiling with an alkali, 14 grs., or 5.18 per cent., a total loss of 9.35 per cent.

There are on the market two brands which are principally sold, and are held at wholesale at the following prices:

| | | | | |
|---|---|---|---|---|
| In 1 pound rolls, | $ .65 | and | $1.12 | per pound. |
| In ½ pound rolls, | .65 | and | 1.35 | per pound. |
| In 1 ounce boxes, | 1.25 | and | —— | per pound. |
| In ½ ounce boxes, | —— | and | 2.25 | per pound. |

Each of the half-ounce boxes contains about 1.45 grs. of cotton.

Good cotton costs at retail about 25 cents per pound; enough alkali to wash it will cost say 10 cents, and clorinated lime 2 or 3 cents; loss in manufacture 10 per cent., which equals 3 cents, making a total cost of material from 40 to 45 cents per pound. This is not counting time, wear of apparatus, fuel, etc. From these data any one can figure out the handsome profit gained by using his leisure moments not otherwise employed in making his own absorbent cotton. The process is simple and easily performed, requires little attention and gives satisfactory results, and if care is taken not to card the cotton or tear it apart from its original carded form, it will, when dry, pull apart readily and look as nice as the commercial samples, except the commercial is recarded and is in one sheet, but otherwise it is its equal. A microscopical examination was made to ascertain the change that takes place in the cotton by this process. Cotton fibres are tubes, with two sides collapsed together, forming ribbon-like fibres; at each edge the fibre does not come wholly together, thus leaving two tubes, one on each side of the fibre. If one end of the crude cotton fibre be brought in contact with water, capillary attraction is generally soon stopped in the tubes by the oil, and no capillary attraction whatever takes place in the fibrous structure itself. After the cotton has been rendered absorbent the fibres will absorb water throughout their whole structure, and the collapsed fibres will distend.

Hence, absorbent cotton is cotton entirely freed from all matter that will obstruct capillary attraction.—FRANK L. SLOCUM, in *American Journal of Pharmacy.*

